# Factors Influencing Adherence to Mammography Screening Guidelines Among Women From Diverse Cultural Backgrounds: A Systematic Review

**DOI:** 10.7759/cureus.116063

**Published:** 2026-09-10

**Authors:** Antonia Christoforidou, Marianna Drakopoulou, Alexandra Mantoudi, Georgia Gerogianni, Marianna Mantzorou, Theodoula Adamakidou, Martha Christoforidou, Paraskevi Apostolara

**Affiliations:** 1 Department of Nursing, University of West Attica, Athens, GRC; 2 Department of Administration, General Hospital “Alexandra", Athens, GRC

**Keywords:** adherence, breast cancer, diverse cultural backgrounds, mammography, migrants

## Abstract

Breast cancer continues to pose a public health challenge as it remains one of the most common malignancies affecting women globally. Although mammography is essential for early detection, adherence to screening guidelines remains unsatisfactory, especially among women from diverse cultural backgrounds. This systematic review aimed to investigate adherence to mammography screening guidelines among women from different cultural backgrounds, evaluate their knowledge, perceptions, and beliefs regarding breast cancer, and additionally identify barriers and facilitators of mammography adherence. A systematic review was conducted in the scientific databases PubMed, Scopus, and ScienceDirect (April-May 2025) for studies published between 2015 and 2025. Fifteen studies (eight quantitative, six qualitative, and one psychometric study) met the predefined inclusion and exclusion criteria. Study selection followed the Preferred Reporting Items for Systematic Reviews and Meta-Analyses (PRISMA) 2020 guidelines, and the methodological quality of the included studies was assessed using Critical Appraisal Skills Programme (CASP) checklists. Higher adherence to mammography screening was associated with higher educational level, health insurance coverage, older age, and advice by a healthcare professional. Major barriers included screening costs, lack of insurance, communication difficulties, cultural and fatalistic beliefs, and feelings of fear and embarrassment. Adherence to mammography screening among women from diverse cultural backgrounds is associated with multiple and complex individual, socioeconomic, and cultural factors. The findings highlight the need for targeted multidimensional and culturally appropriate interventions to reduce inequalities and increase participation rates in breast cancer screening.

## Introduction and background

Breast cancer is the most commonly diagnosed cancer among women worldwide and remains the leading cause of cancer-related death [1]. According to the Global Cancer Observatory (GLOBOCAN) data, approximately 2.3 million new cases and an estimated 670,000 deaths were reported globally in 2022 [2]. This high incidence is associated with an estimated lifetime risk of approximately one in eight women [3-5].

Significant inequalities exist in the global burden of breast cancer according to the Human Development Index (HDI). In high-HDI countries, approximately one in 12 women will be diagnosed with breast cancer, while one in 71 women will die from the disease. In contrast, in low-HDI countries, one in 27 women will be diagnosed with breast cancer, and one in 48 women will die from the disease [2].

Similarly, survival rates also differ substantially, approaching or exceeding 90% in high-income countries but remaining lower in low- and middle-income countries, as observed in India (66%) and South Africa (40%) [2]. These differences reflect inequalities in access to healthcare services, early detection, and treatment between developed and developing countries [6].

These findings highlight the importance of strengthening breast cancer prevention through systematic screening programs and the implementation of interventions related to health promotion and health education. As part of secondary prevention, these programs mainly include mammography as the primary population-based screening tool. In addition, breast self-examination (BSE), clinical breast examination (CBE), breast ultrasound, and magnetic resonance imaging (MRI) of the breast are also used [7], whereas their indications vary according to individual risk factors and national guidelines.

However, mammography is the main screening method for breast cancer prevention, supported by international guidelines from the World Health Organization (WHO) [8], the United States Preventive Services Task Force (USPSTF) [9], and the European Commission Initiative on Breast Cancer (ECIBC) [10]. Although these guidelines aim to promote early detection in asymptomatic women, screening recommendations differ among countries, as they are determined by national health authorities and adapted to age and risk factors of the target population.

Women's adherence to screening guidelines has been widely investigated. Women from migrant or culturally diverse backgrounds, who may represent a vulnerable social group, show low participation in mammography screening because of barriers such as communication difficulties, limited access, and cultural beliefs. Although many studies have examined adherence, perceptions, and barriers to screening, the available evidence remains fragmented across individual studies, making it difficult to identify consistent factors associated with adherence and develop culturally appropriate interventions.

This systematic review provides a comprehensive synthesis of studies published between 2015 and 2025, focusing on breast cancer prevention among women from migrant or culturally diverse backgrounds. Unlike previous reviews, the present systematic review has a broader population and geographical scope while focusing specifically on mammography screening. Nguyen et al. [11] focused exclusively on Vietnamese American women and examined breast cancer screening, diagnosis, and outcomes, whereas Nassur et al. [12] focused on refugees resettled in the United States and examined barriers to breast, cervical, and colorectal cancer screening. In contrast, the present review includes women from diverse cultural backgrounds across multiple geographical settings and synthesizes evidence specifically on mammography adherence, knowledge, perceptions and beliefs, as well as barriers and facilitators to screening.

This systematic review aims to investigate adherence to mammography screening guidelines among women from diverse cultural backgrounds compared with native populations, examine knowledge, perceptions, and beliefs regarding breast cancer and its prevention, identify culturally related barriers affecting mammography uptake, and highlight practical and policy interventions that could improve adherence and participation in breast cancer screening.

## Review

Methodology

Search Strategy

This systematic review was conducted from April to May 2025. A systematic search was performed in PubMed, Scopus, and ScienceDirect for studies published between January 1, 2015, and May 7, 2025, with the final literature search conducted on May 7, 2025. These sources were selected to provide broad coverage of biomedical and health sciences literature relevant to breast cancer screening and culturally diverse populations; no additional searches of gray literature or reference lists were conducted. Predefined keywords and Boolean operators were used to identify the most relevant studies. The search queries used in PubMed, Scopus, and ScienceDirect are presented in Table 1.

**Table 1:** Search queries.

Database	Search date	Query	Filters	Search results
PubMed	April 30, 2025	(Breast cancer screening OR mammography OR Guidelines adherence OR Screening compliance OR Knowledge and cancer prevention OR screening attitudes OR screening beliefs) AND (Cultural barriers in screening OR Barriers to screening participation OR Preventive healthcare disparities) AND (Ethnic minorities and breast cancer OR Migrant women and cancer screening OR native women).	Female adults: 19+ years, 2015-2025	86
Scopus	May 04, 2025	("breast cancer screening" OR "mammography") AND ("migrant women" OR "ethnic minorities" OR "native women" OR "immigrants") AND ("cultural barriers" OR "breast screening barriers" OR "preventive healthcare disparities") AND ("breast cancer attitudes" OR "breast cancer beliefs" OR "breast screening beliefs" OR "breast screening attitudes" OR "breast screening perception" OR "breast cancer prevention knowledge").	Female, English, 2015-2025	12
Science Direct	May 07, 2025	("breast cancer screening" OR "mammography") AND ("immigrant women" OR "migrants" OR "women") AND ("screening beliefs" OR "screening attitudes" OR "screening perception" OR "cancer prevention knowledge" )	2015-2025	75

Eligibility Criteria

Studies were selected based on inclusion and exclusion criteria, which concerned the target population, type of studies, language, and year of publication. The inclusion criteria included studies involving native women and women from diverse cultural backgrounds aged over 18 years, focusing on adherence to mammography screening, barriers to participation in screening, and knowledge, attitudes, and perceptions regarding breast cancer and its prevention.

For the purposes of this review, “women from diverse cultural backgrounds” referred to migrant or immigrant women and women belonging to cultural, ethnic, or religious minority groups within the country where the study was conducted. This criterion was applied during study selection based on the population characteristics explicitly reported by the authors of each study.

Eligible study designs included qualitative and quantitative observational studies, such as cross-sectional and prospective studies, as well as methodological (psychometric) studies assessing health belief instruments. Mixed-methods studies were also eligible provided that they met the remaining predefined inclusion criteria; however, no mixed-methods studies were included in the final review.

The exclusion criteria included systematic literature reviews, interventional studies, studies not published in English, studies published outside the predefined period (2015-2025), and studies including only native women without comparative reference to migrant or culturally diverse women. Interventional studies were excluded because this review aimed to synthesize factors associated with mammography adherence under non-interventional conditions rather than to evaluate changes in screening behavior resulting from specific interventions.

PRISMA Flow Diagram

The selection of studies that were eventually included in the systematic review was performed through a multi-stage screening process to determine study eligibility according to the predefined inclusion and exclusion criteria. The study selection process comprised identification, title and abstract screening, full-text eligibility assessment, and final inclusion. This process is illustrated in the PRISMA 2020 (Preferred Reporting Items for Systematic Reviews and Meta-Analyses) flow diagram presented in Figure 1.

**Figure 1:**
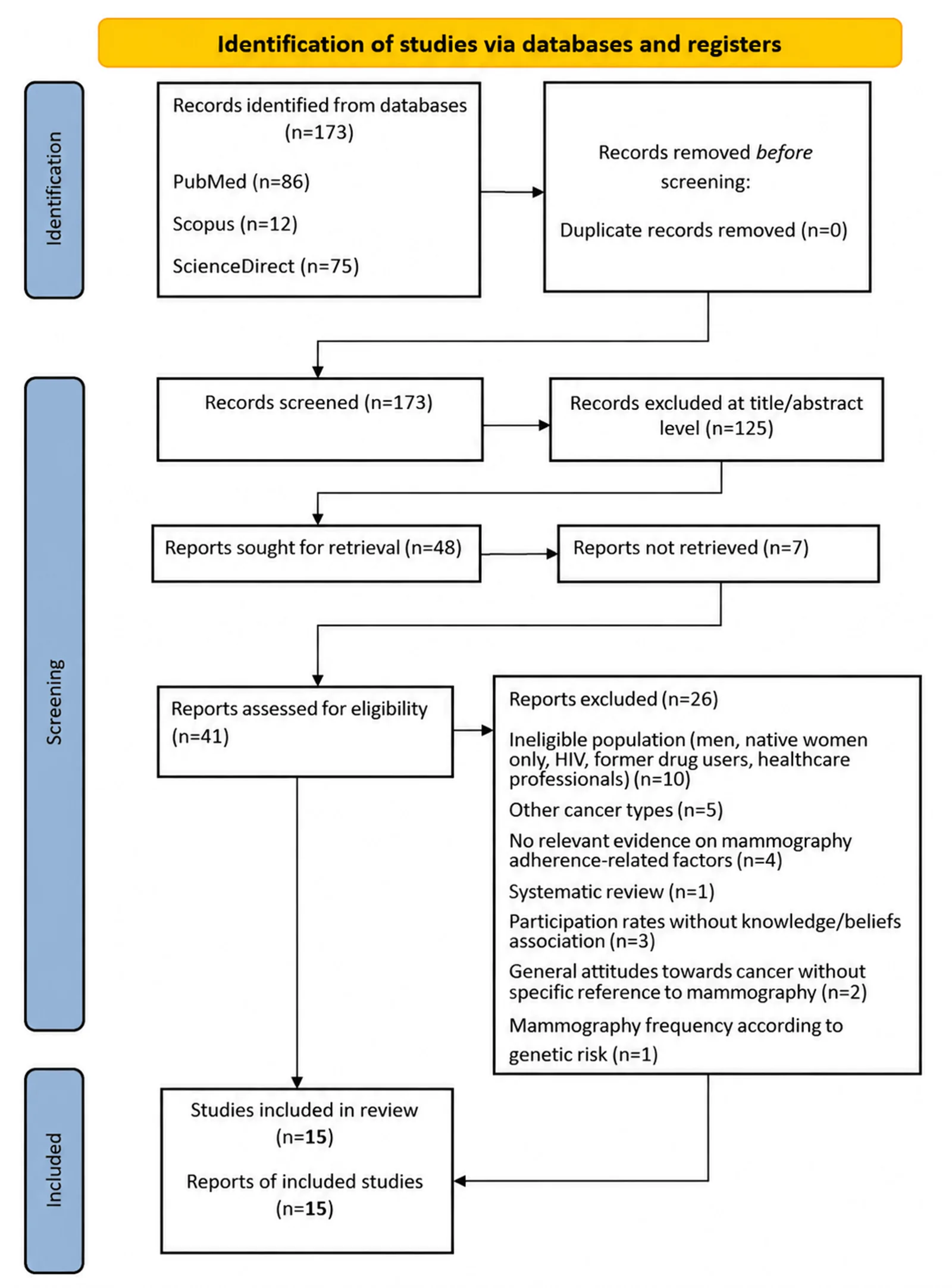
PRISMA 2020 flow diagram of the study selection process. PRISMA, Preferred Reporting Items for Systematic Reviews and Meta-Analyses

Initially, 173 records were identified from the databases (PubMed n = 86, Scopus n = 12, ScienceDirect n = 75), using the predefined keywords and Boolean operators. Duplicate records were assessed manually by cross-checking article titles and author names across all the records from PubMed, Scopus, and ScienceDirect. As no duplicate records were identified, all 173 records proceeded to screening.

The predefined inclusion and exclusion criteria were applied throughout the title and abstract screening and the full-text eligibility assessment. At the first stage, titles were screened to exclude records that were not relevant to the topic of the present review. At this stage, 95 records were excluded, while 78 records were retained for abstract screening. An additional 30 records were excluded (125 records excluded in total during title and abstract screening), while 48 records remained and proceeded to the next stage..

This was followed by the retrieval of the full-text reports. At this stage, 7 reports could not be retrieved. Consequently, 41 reports were retained for the final full-text assessment for eligibility.

During the full-text screening stage, 26 reports were excluded. Ultimately, 15 studies met the predefined inclusion criteria and were included in the systematic review. This study selection was performed independently by two reviewers. Any disagreements between the two reviewers were resolved through discussion and consensus.

Reasons for Full-Text Exclusion

The reasons for exclusion at this final stage were the reference to general attitudes toward cancer without specific reference to mammography (*n* = 2), the absence of relevant evidence on factors associated with mammography adherence or on knowledge, beliefs, perceptions, barriers, or facilitators related to mammography screening (*n* = 4), the target population that did not meet the inclusion criteria: inclusion of men (*n* = 2), specific subgroups (former drug users, women with HIV) (*n* = 2), inclusion of only native women without comparison with migrant women (*n* = 4), reference to other forms of cancer besides breast cancer (e.g., cervical, colorectal) (*n* = 5), a systematic review article (*n* = 1), articles referring to healthcare professionals’ adherence to screening guidelines (*n* = 2), reference only to participation rates in mammography without associations with knowledge or beliefs (*n* = 3), and reference to women's attitudes toward modifying mammography frequency based on genetic risk (*n* = 1).

Critical Appraisal of Included Studies

The methodological quality of the included studies was assessed using the Critical Appraisal Skills Programme (CASP) checklists. According to the study design, different tools were applied (qualitative or quantitative), establishing a systematic evaluation of methodological quality and potential bias. One included study was a methodological (psychometric) study assessing the validity and reliability of a screening-related instrument and was not appraised using a CASP checklist, as CASP checklists are not designed for psychometric studies. No alternative formal quality appraisal tool was applied to this study; therefore, its methodological quality was not formally assessed.

Overall, of the 14 studies assessed using the CASP checklists, four studies were assessed as high quality and 10 as moderate quality, while no studies were classified as low quality. More specifically, among the quantitative studies, two were assessed as high quality and six as moderate quality, while among the qualitative studies, two were assessed as high quality and four as moderate quality.

The main methodological limitations contributing to the moderate quality ratings of several studies included the use of self-reported data, limited generalizability, and insufficient reporting of the relationship between researchers and participants in some qualitative studies. The methodological quality assessment was used to provide an overall evaluation of the strength and limitations of the included evidence. Studies were not excluded or formally weighted according to their methodological quality during the narrative synthesis, and findings from all eligible studies were considered in the interpretation of the results.

Data Synthesis

Due to differences in study design, populations, and outcomes among the included studies, a meta-analysis was not performed. The findings were narratively synthesized and grouped into three main categories: mammography screening participation, factors associated with greater adherence, and barriers associated with lower adherence. No formal framework was used for the narrative synthesis.

Results

Characteristics of Included Studies

The systematic review included a total of 15 studies that met the predefined inclusion criteria. The eligible studies were published in the predefined period 2015-2025, and the target population consisted of native women and women from diverse cultural backgrounds aged over 18 years. The studies were conducted on three continents. Most of the eligible studies were conducted in America (United States *n* = 6, Canada *n* = 1), five in Australia, and three in Europe and the Middle East (Scotland, Spain, and Israel).

Of the 15 studies, 8 were cross-sectional quantitative observational studies using structured questionnaires and statistical analyses, 6 studies had a qualitative design conducted through personal interviews, and one focused on the psychometric validation of a breast cancer screening beliefs questionnaire. Sample sizes ranged from 11 to 65 participants in the qualitative studies and from 98 to 799 participants in the quantitative observational studies, while the psychometric validation study included 251 participants.

One study included native women (i.e., those originating from the country where the study was conducted), located in Australia. Twelve studies investigated migrant women living in host countries, such as Canada (*n* = 1), the United States (*n* = 6), Scotland (*n* = 1), and Australia (*n* = 4). The remaining two studies, conducted in Europe, compared native and migrant women in Spain and two different religious groups in Israel.

The research tools used varied across the included studies and aimed to assess women's knowledge, attitudes, and perceptions regarding adherence to cancer prevention guidelines and mammography screening. Selected studies were based on theoretical models, such as the Health Belief Model, or used validated instruments, such as Champion's Health Belief Model Scale of Mammography Screening and Kwok's Breast Cancer Screening Beliefs Questionnaire, which assesses cultural beliefs, attitudes, and perceived barriers related to adherence.

Systematic Review’s findings

The findings of the studies focused on three basic topics: women’s participation rates in screening, facilitators of adherence to mammography screening, and barriers to mammography screening.

Screening participation: Participation in screening and women’s adherence to mammography varied across studies. These variations may be related to differences in study populations, healthcare systems, access to screening services, and the organization of screening programs across the countries where the studies were conducted.

As the following participation rates were derived from independent studies with different study designs, sampling approaches, and healthcare settings, they should be interpreted within the context of each study rather than as directly comparable estimates across cultural groups.

In studies conducted in North America (United States and Canada), 88% of African migrants [13], 66.2% of American Indian women [14], and 87% of Asian migrants [15] reported having undergone a mammogram at least once. In Europe (Spain), a comparative study between migrant women from low-income countries and native women of similar low socioeconomic status revealed a lack of interest in disease prevention and screening, as well as a reluctance to talk about cancer, regardless of country of origin [16].

Similarly, in Israel, Arab women showed greater adherence to mammography compared with Jewish ultra-Orthodox women [17]. However, the study also identified differences in health beliefs and barriers between the two groups. Higher mammography adherence was associated with lower levels of religious beliefs and higher perceived cancer risk, while the absence of a physician’s recommendation was associated with lower adherence [17].

In Australia, 23.6% of Indian-Australian women within the targeted age group reported undergoing mammography every two years [18], while nearly two-thirds of African migrants reported having undergone this screening [19]. In addition, among Arabic-Australian women, the frequency of mammography participation was associated with attitudes toward general health check-ups and perceived barriers to mammographic screening [20].

Among Aboriginal women in Western Australia, participation in mammography screening was lower than that of the overall female population in Western Australia. The study identified limited knowledge and understanding of screening, cultural beliefs, and competing health and life priorities as barriers to participation [21]. Among Thai migrant women in Australia, women reported limited use of mammography screening services, with language difficulties, unfamiliarity with the healthcare system, and limited accessibility of screening services reported as barriers [22].

Facilitators of adherence: Factors associated with greater adherence, according to the findings of the studies, are presented below. One of the most frequently reported factors positively associated with mammography participation was a higher level of education, which was reported in six included studies.

Women with higher educational attainment tended to have more knowledge about breast cancer and recognized the importance of early diagnosis. At the same time, higher educational level tended to be associated with greater perceived cancer risk, greater perceived benefits of prevention, greater screening awareness, and higher adherence [13,15-18,20].

Health insurance coverage was associated with higher mammography participation in two included studies [13,23].

Findings from other studies (*n* = 3) showed that recommendations from healthcare professionals, informational intervention programs, and appointment reminders were associated with greater adherence to screening [17,21,23]. In addition, factors such as a supportive family environment and social support contributed to a more positive attitude toward prevention (*n* = 2) [18,21].

Other studies have reported higher adherence among older women (*n* = 2) [14,19] and married women (*n* = 2) [13,18]. Furthermore, previous experience with mammography, which facilitates navigation of the healthcare system, as well as knowing someone diagnosed with breast cancer, which strengthens concern for personal health and well-being (*n* = 2), were also associated with higher adherence [15,24]. Overall, these factors were associated with greater awareness, more positive attitudes toward prevention, and higher mammography adherence.

Barriers to mammography screening: Several factors were reported as barriers to adherence to mammography screening. One of the most frequently reported barriers in the included studies (*n* = 7) was the cost of screening, either in combination with the lack of health insurance coverage or independently of it. Financial difficulties and limited economic resources make it difficult for women to cover required expenses, including transportation costs and the cost of screening itself, thereby limiting their access to healthcare services [13,17,19,22-25].

Another frequently reported barrier (*n* = 7) was fear, both of the examination itself and of its possible result. The unfamiliar procedure, the perception that the examination may be painful, as well as the possibility of a positive diagnosis reinforced fear, which may deter them from participating in preventive screening [14,16,17,20,21,25,26].

Shame and embarrassment during the examination, especially when the doctor is male, are also reported as barriers (*n* = 5) [14,20-22,26]. The included studies also reported cultural beliefs and fatalistic perceptions of cancer as barriers to mammography screening (*n* = 6). Differences in health culture, conflicts between cultural values, the perception that mammography is unnecessary for asymptomatic women, strong religious beliefs about health and illness, as well as perceptions and misconceptions about breast cancer, were associated with non-adherence [17,20,21,23,25,27].

Similarly, language barriers reported in the included studies were associated with non-adherence (*n* = 8). On the one hand, the inability to communicate in their native language and difficulties in understanding English due to limited language proficiency may restrict access to healthcare services. On the other hand, the absence of culturally adapted interventions may result in limited information and awareness in women’s native language about breast cancer and its prevention [16,19-23,25,27].

In addition, some studies (*n* = 5) reported competing personal and family responsibilities as barriers to screening, including lack of time, childcare, family responsibilities, and work commitments [14,16,17,25,26]. Finally, differences in healthcare systems (*n* = 2) [22,26], insufficient or incorrect information about prevention (*n* = 1) [21], and low levels of knowledge about breast cancer (*n* = 3) [22,25,26] were additional factors associated with lower participation. The detailed results of the methodological quality assessment are presented in Tables 2-3.

**Table 2:** Methodological quality appraisal of the included quantitative studies using the CASP checklist. CASP, Critical Appraisal Skills Programme

Section A: Are the results valid?		Sheppard et al., 2015 [13]	Kwok et al., 2015 [18]	Hippman et al., 2016 [15]	Ogunsiji et al., 2017 [19]	Roh et al., 2018 [14]	Freund et al., 2019 [17]	Miller et al., 2020 [24]	Gill et al., 2023 [23]
1	Did the study address a clearly focused issue?	Yes	Yes	Yes	Yes	Yes	Yes	Yes	Yes
2	Did the authors use an appropriate method to answer their question?	Yes	Yes	Yes	Yes	Yes	Yes	Yes	Yes
3	Were the subjects recruited in an acceptable way?	Yes (convenience sample)	Yes (convenience sampling)	Yes (clinic-based sample)	Yes (community-based sample)	Yes (convenience sample)	Yes(convenience sample)	Yes (convenience sample)	Yes (convenience sample)
4	Were the measures accurately measured to reduce bias?	Yes (self-reported data)	Yes (self-reported, validated questionnaire)	Yes (self-reported data)	Yes (self-reported data)	Yes (self-reported data, validated tools)	Yes (self-reported data)	Yes (self-reported data, validated scales)	Yes (self-reported data)
5	Were the data collected in a way that addressed the research issue?	Yes	Yes	Yes	Yes	Yes	Yes	Yes	Yes
6	Did the study have enough participants to minimize the play of chance?	Yes (moderate sample size)	Yes (moderate sample size)	Yes (moderate sample size)	Yes (moderate sample size)	Yes (moderate sample size)	Yes (large sample size)	Yes (moderate sample size)	Yes (large sample size)
7	How are the results presented, and what is the main result?	Clearly presented	Clearly presented	Clearly presented	Clearly presented	Clearly presented	Clearly presented	Clearly presented	Clearly presented
8	Was the data analysis sufficiently rigorous	Yes	Yes	Yes	Yes	Yes	Yes	Yes	Yes
9	Is there a clear statement of findings?	Yes	Yes	Yes	Yes	Yes	Yes	Yes	Yes
10	Can the results be applied to the local population?	Yes (limited generalizability)	Yes (with caution)	Yes (limited generalizability)	Yes (limited generalizability)	Yes (with caution)	Yes (limited generalizability)	Yes (limited generalizability)	Yes (with caution)
11	How valuable is the research?	Yes	Yes	Yes	Yes	Yes	Yes	Yes	Yes
	Overall methodological quality	Moderate	Moderate	Moderate	Moderate	Moderate	High	Moderate	High

**Table 3:** Methodological quality appraisal of the included qualitative studies using the CASP checklist. CASP, Critical Appraisal Skills Programme

Section A: Are the results valid?		Saadi et al., 2015 [26]	Pilkington et al., 2017 [21]	March et al., 2018 [16]	Gorman et al., 2018 [27]	Suwankhong et al., 2018 [22]	Kohler et al., 2025 [25]
1.	Was there a clear statement of the aims of the research?	Yes	Yes	Yes	Yes	Yes	Yes
2.	Is a qualitative methodology appropriate?	Yes	Yes	Yes	Yes	Yes	Yes
3.	Was the research design appropriate to address the aims of the research?	Yes	Yes	Yes	Yes	Yes	Yes
4.	Was the recruitment strategy appropriate to the aims of the research?	Yes	Yes	Yes	Yes	Yes	Yes
5.	Was the data collected in a way that addressed the research issue?	Yes	Yes	Yes	Yes	Yes	Yes
6.	Has the relationship between researcher and participants been adequately considered?	Yes	Can’t tell	Can’t tell	Can’t tell	Can’t tell	Yes
Section B: What are the results?							
7.	Have ethical issues been taken into consideration?	Yes	Yes	Yes	Yes	Yes	Yes
8.	Was the data analysis sufficiently rigorous?	Yes	Yes	Yes (not extensively detailed)	Yes	Yes	Yes
9.	Is there a clear statement of findings?	Yes	Yes	Yes	Yes	Yes	Yes
Section C: Will the results help locally?							
10.	How valuable is the research?	Yes	Yes	Yes	Yes	Yes	Yes
Overall methodological quality		High	Moderate	Moderate	Moderate	Moderate	High

Overall, the findings suggest that adherence to mammography screening was associated with a wide range of educational, economic, social, and cultural factors. The main characteristics of the included studies in the systematic review are presented in Table 4.

**Table 4:** Main characteristics of studies included in the systematic review.

A/A	Author/year	Population/origin	Study design	Sample	Aim	Main findings
1	Sheppard et al., 2015 [13]	African immigrant women	Cross-sectional quantitative study	200	The aim of this study was to examine factors associated with endorsement of breast cancer screening practices among women of African origin.	Overall participation in breast cancer screening was high (81%). The proportion of women who had ever undergone a mammogram was 88%, which was higher than findings from other studies involving African immigrant populations (15%-61%). Factors significantly associated with higher adherence to screening included greater breast cancer knowledge, English as a primary language, marital status (being married), and having healthcare insurance. Reported barriers limiting access to healthcare services included lack of insurance coverage and the cost of examinations.
2	Saadi et al., 2015 [26]	Bosnian, Iraqi, and Somali women refugees	Qualitative study	57	The aim of this study was to explore refugee women’s beliefs regarding preventive healthcare and breast cancer screening.	Women across all groups expressed willingness to participate despite systemic and personal barriers and fears. Key facilitators included access to someone who spoke their language and could explain procedures, as well as recommendations, reminders, and scheduling help from health providers. Many participants described psychosocial barriers to screening uptake, such as fear of pain and modesty-related concerns. Additional barriers reported by women included work and childcare responsibilities, financial difficulties, and cultural and fatalistic beliefs. Differences were observed among groups: Somali women had a more limited understanding of preventive care and mammography, Iraqi women were relatively more knowledgeable, and approximately 70% of Bosnian women reported willingness to attend annual checkups.
3	Kwok et al., 2015 [18]	Indian-Australian women	Cross-sectional quantitative study	242	The aim of the study was to examine the relationship between demographic characteristics, cultural beliefs, and women's breast cancer screening behaviors.	The majority of participants (72.7%-81.4%) had heard of breast awareness, clinical breast examination, and mammography. Only 23.6% of women within the targeted age group reported undergoing mammography biennially. Facilitators included longer residence in Australia, marital status (being married), and family support. Barriers included cultural beliefs and perceptions, misconceptions about breast cancer, lack of awareness, and low perceived susceptibility. Women who engaged in the recommended screening practices had significantly more positive attitudes toward general health checkups and reported fewer perceived barriers to mammographic screening.
4	Hippman et al., 2016 [15]	Asian immigrant women attending the Asian Women’s Health Clinic in Canada	Cross-sectional quantitative study	98	The purpose of this study was to assess mammography uptake, knowledge, beliefs, and predictors (barriers and facilitators) of mammography screening.	Eighty-seven percent of women had undergone mammography at least once, while 13% had never undergone mammography. Participants’ knowledge related to breast cancer was quite low, although they were generally highly educated. Predictors of screening were lower perceived barriers to mammography and knowing someone who had had breast cancer, while greater perceived benefits of early detection and treatment of breast cancer were marginally associated with mammography uptake.
5	Kwok et al., 2016 [20]	Arabic-Australian women	Quantitative psychometric validation study	251	To report the psychometric properties of the Arabic version of the Breast Cancer Screening Beliefs Questionnaire.	The Arabic version of the BCSBQ had satisfactory validity and internal consistency (Cronbach’s α = 0.81-0.93). The frequency of breast cancer screening practices, including mammography, was significantly associated with attitudes toward general health checkups and perceived barriers to mammographic screening. The Arabic version of the BCSBQ was found to be a culturally appropriate, valid, and reliable instrument for assessing beliefs, knowledge, and attitudes toward breast cancer and breast cancer screening practices among Arabic-Australian women.
6	Pilkington et al., 2017 [21]	Aboriginal women in Western Australia	Qualitative study	65	This study aimed to explore factors influencing participation in mammography screening among Aboriginal women in Western Australia and to identify potential strategies to increase participation.	Many participants expressed willingness to participate in breast cancer screening and emphasized the importance of accessible and culturally appropriate services. Factors perceived as potential facilitators of participation included a sense of personal investment in health and well-being, health education, and information and support groups. Identified barriers to mammography screening included lack of awareness about screening, limited cultural appropriateness of services, cultural and fatalistic beliefs, fear of diagnosis, and feelings of shame or embarrassment during the examination.
7	Ogunsiji et al., 2017 [19]	African migrant women	Descriptive cross-sectional study	264	The aim of this study was to examine factors associated with breast cancer screening behaviour and screening status among African migrant women in Australia.	The majority of the women had heard of breast awareness (76.1%) and mammography (85.2%), although only 11.4% practiced breast awareness monthly. Nearly two-thirds of the participants (65%) had undergone mammography. Region of origin within the African continent was significantly associated with breast cancer screening behavior. Older age and employment status were significantly associated with higher participation in mammography. Reported barriers included transportation difficulties, cost, and language barriers.
8	March et al., 2018 [16]	Migrant women from low-income countries and native Spanish women of similar low socioeconomic status	Qualitative study	36	The aim of this study was to explore knowledge, perceptions, and barriers related to breast cancer screening among migrant women from low-income countries and native Spanish women of similar low socioeconomic status.	Many women expressed low interest in disease prevention in general and cancer prevention in particular. Many women were generally reluctant to discuss cancer or receive related information. Although they acknowledged the importance of early diagnosis, breast cancer was frequently associated with death. Perceptions of prevention ranged from preventive to fatalistic attitudes, shaping women’s attitudes toward screening. Common barriers included fear of the screening result and lack of time. Additional barriers among migrant women included language barriers, frequent mobility, and concerns related to legal or immigration status.
9	Gorman et al., 2018 [27]	Polish migrants to Scotland	Qualitative study	11	The aim of the study was to explore factors influencing Polish migrants' participation in the national breast screening program in Scotland and to identify improvements that could increase informed participation.	Polish women often used both the Polish and Scottish healthcare systems. Polish women reported lower screening uptake. Barriers to adherence included limited knowledge of the healthcare system, cultural differences in prevention, fear, language issues, the absence of information in Polish, work commitments, and family responsibilities.
10	Roh et al., 2018 [14]	American Indian women	Cross-sectional quantitative study	286	The aim of the study was to examine health beliefs and breast cancer literacy and their association with breast cancer screening among American Indian women.	The majority of women were aware of clinical breast examination (CBE) (91.5%) and mammograms (94.3%) and reported moderate breast cancer literacy, while 75.0% had undergone a CBE and 66.2% had undergone a mammogram. Greater knowledge of breast cancer and perceived benefits of screening were not significant predictors of CBE and/or mammography uptake. Perceived barriers, such as fear of diagnosis, lack of understanding of the screening procedure, embarrassment, time constraints, and anticipated pain, were significantly associated with lower participation in both CBE and mammograms. Older age and being divorced or widowed were significant determinants of screening participation.
11	Suwankhong et al., 2018 [22]	Thai migrant women	Qualitative study	25	The aim of this study was to explore barriers to breast cancer screening programs among Thai migrant women in Australia.	Most Thai migrant women demonstrated low perceived susceptibility to breast cancer, which reduced their perceived need and motivation for screening and limited their participation in breast cancer prevention programs. Identified barriers to mammography screening included limited knowledge and information about breast cancer, differences in the healthcare system in Australia, modesty-related concerns such as embarrassment and shame during examination, and language barriers.
12	Freund et al., 2019 [17]	Jewish ultra-Orthodox women and Arab women in Israel	Cross-sectional quantitative study	n = 398 Jewish ultra-Orthodox women; n = 401 Arab women	This study examined factors associated with adherence to breast cancer screening in two distinct groups in Israel: Arab women representing a cultural-ethnic minority and Jewish ultra-Orthodox women representing a cultural-ethnic faith-based minority.	Arab women demonstrated higher adherence to both mammography and clinical breast examination (CBE) compared with Jewish ultra-Orthodox women. Jewish ultra-Orthodox women reported stronger religious beliefs, higher levels of modesty-related concerns, and higher perceived susceptibility to breast cancer. Arab women reported greater social and access-related barriers, as well as higher perceived severity of breast cancer. Higher perceived susceptibility and greater fear of cancer-related losses were positively associated with adherence to both mammography and CBE in both groups. Strong religious beliefs were negatively associated with adherence only among Jewish ultra-Orthodox women. Religious beliefs and fear of cancer-related losses were identified as significant predictors of screening adherence.
13	Miller et al., 2020 [24]	Chinese American women (who had not had a mammogram in the past 12 months)	Cross-sectional quantitative study	128	To examine psychosocial barriers associated with mammography screening history between women who were currently or ever nonadherent and women who had never had a mammogram.	Women who reported a greater lack of access and lack of need were less likely to have ever had a mammogram. Women who reported greater modesty were more likely to have ever had a mammogram. Modesty was associated with previous mammography experience.
14	Gill et al., 2023 [23]	Women with non-English language preference (NELP)	Cross-sectional quantitative study	420	The aim of this study was to examine barriers, facilitators, perceptions, and communication-related factors associated with breast cancer screening among women with non-English language preference (NELP).	The most significant demographic difference between women who had undergone mammography and those who had not was insurance coverage, highlighting potential financial or structural barriers to access. Perceptions and beliefs regarding the need for mammography were associated with lower screening uptake. Provider communication, including recommendation of mammography and discussion of benefits and risks, was significantly associated with a higher likelihood of screening participation. Reported reasons for missing screening included absence of symptoms or concerns, the COVID-19 pandemic, fear, anticipated pain, previous negative experience, personal choice, forgetfulness, and being busy.
15	Kohler et al., 2025 [25]	South Asian immigrant women in New Jersey	Qualitative study	22	To explore perceptions and beliefs regarding breast cancer risk and mammography screening, as well as factors influencing adherence to screening.	Women demonstrated awareness of breast cancer risk and screening benefits; however, fears, low perceived susceptibility to breast cancer, and misconceptions persisted (e.g., wearing a bra increases cancer risk). Cultural values often prioritized family obligations over personal health, influencing adherence. Barriers included stigma, modesty, cost, insurance limitations, and language barriers limiting access to screening.

Discussion

The present systematic review indicates that women from diverse cultural backgrounds exhibit varying levels of adherence to mammography screening. Adherence was associated with a complex interplay of socioeconomic, cultural and individual factors. The findings identify both facilitators and barriers, highlighting the multifaceted nature of screening and suggesting that healthcare behavior is shaped not only by individual knowledge and attitudes but also by broader social and structural factors.

In this systematic review, the term *adherence* refers to attendance in mammography according to applicable and updated guidelines, while the populations described as being from *diverse cultural backgrounds* included migrant or immigrant women and women belonging to cultural, ethnic, or religious minority groups. This definition was prespecified in the eligibility criteria and consistently applied during study selection based on the population characteristics explicitly reported by the authors of each study. It should be also mentioned that although one study focused on the psychometric validation of a questionnaire for breast screening beliefs and not for screening adherence, it provided valuable insights into the cultural beliefs and perceived barriers, which play a central role in screening behavior [20].

Certain demographic and healthcare-related characteristics, such as level of education, marital status, age, access to healthcare services, healthcare insurance coverage, and the role of healthcare professionals, are identified as factors associated with greater mammography adherence.

Higher educational level was reported as a factor associated with mammography adherence. Women with higher levels of education are likely to demonstrate greater health literacy and are more accepting of health information, recognizing the importance of prevention. This finding is also supported by studies that highlight the role of health literacy in enhancing adherence. Across different geographical regions, early studies conducted in Italy [28] and Bangladesh [29], as well as a more recent study from Iran [30] and systematic reviews [31], have also reported associations between higher educational levels, increased awareness and breast cancer screening adherence.

Healthcare insurance coverage was also associated with greater mammography participation in the included studies. This association may reflect differences in access to healthcare services and screening-related costs. Lack of insurance coverage was also associated with reduced participation, suggesting that social inequalities and financial barriers may be related to limited access to prevention, even in developed countries.

This finding from the present review is also supported by the broader literature, reinforcing the role of structural and financial determinants. Similar associations have been reported in primary studies by Abdou et al. [32], Halmata et al. [33], Elias-Rizk et al. [34], and Chandra et al. [35], including women from Nigeria, Cameroon, and Lebanon, as well as employed women in Texas, and in reviews by Racine et al. [36] and Ponce-Chazarri et al. [37], including Arab Muslim migrant and refugee women and vulnerable populations.

Another important factor positively associated with mammography uptake is the recommendation and communication from healthcare professionals. Similar conclusions have been reported in other studies [38-41]. This finding suggests that culturally sensitive communication and appropriate intercultural approaches by healthcare professionals may help build trust and promote more positive attitudes toward prevention.

Higher adherence was observed among married and older women. Possible explanations may include a greater sense of responsibility for maintaining health and encouragement from their spouses. Similar findings have been reported by Tavakoli et al. [41], Omisore et al. [42], and Ponce-Chazarri et al. [37]. On the other hand, in the broader literature, Mohammadnabizadeh et al. [30] reported that older women demonstrate a higher perceived need for prevention, likely due to increased prevalence of health problems over time and more frequent interaction with healthcare services, which facilitates a better understanding of the healthcare system. However, conflicting findings have been reported regarding the association between mammography uptake and marital status and age [30,39].

Financial barriers were consistently associated with lower mammography adherence, particularly among populations consisting of women from vulnerable social groups with low socioeconomic status. In contrast to the facilitating role of insurance coverage, the absence of health insurance and the cost of the examination combined with limited financial resources may represent barriers to accessing healthcare services. This finding from the present review is also supported by the broader literature, as financial inequalities in access are observed both in Western countries, such as in the study by Rauch et al. conducted in the USA and including African migrant women [43], and in Middle Eastern countries, such as Saudi Arabia [44], as well as among populations of Asian origin [45]. Based on these findings, improving the accessibility of breast screening programs for women regardless of their country of origin may represent an important public health strategy to reduce inequalities in screening.

Another inhibiting factor to mammography screening adherence is language barriers. Difficulties in communication and understanding, insufficient information in women’s native language, and the absence of culturally competent interventions may deter women from accessing healthcare services and impede the adoption of preventive behaviors. These findings from the present review are consistent with the broader literature. Similar language and communication barriers have been reported in primary studies by Bhargava et al., who examined Pakistani migrant women in Norway [46], and Jamal et al., who focused on culturally diverse women in Sydney [40], as well as in systematic reviews by Racine et al. [36], Tavakoli et al. [41], and Dumky et al. [47].

In addition, cultural and religious beliefs were consistently associated with women’s attitudes toward prevention. Fatalistic beliefs, strong religious perceptions about disease and illness, and misconceptions about cancer were reported as barriers to mammography screening in the present review. These findings are also supported by the broader literature. Racine et al. [36], Tavakoli et al. [41], Rauch et al. [43], and Sathiyaseelan et al. [48] reported religious beliefs and fatalism as barriers to mammography screening.

Another significant finding pertains to the psychological factors that inhibit adherence. Deterrents to mammography uptake may be fear of the actual examination, fear of diagnosis, pain concerns during the examination, and feelings of shame and embarrassment related to breast exposure and discomfort in the presence of a male doctor. These findings are consistent with the broader literature, including studies conducted by Alanazi et al. in Saudi Arabia [49], by Bao et al. in China [50], as well as with the reviews by Sathiyaseelan et al. [48] and Ebrahimoghli et al. [31]. From a practical perspective, these findings suggest that providing appropriate health information and improving communication with women may help address fear and support mammography adherence.

Regarding participation rates, the observed differences may be related to variations in healthcare systems, study populations, and screening contexts across the countries where the studies were conducted. In the United States and Canada, relatively high participation rates in mammography screening were reported among several populations included in the reviewed studies, such as African immigrant women, American Indian women, and Asian immigrant women [13-15].

This finding appears to differ from the conclusions of Ponce-Chazarri et al., who reported greater barriers to adherence among Black, Hispanic, and Asian women [37]. However, this apparent discrepancy may reflect differences in healthcare systems, study populations, and screening policies and guidelines across settings. Furthermore, a greater number of reported barriers does not necessarily translate into lower participation rates, particularly in countries where free screening programs and insurance coverage are available.

Among women from diverse cultural backgrounds, differences in mammography participation were also observed depending on country of origin and geographical settings [18-20]. These findings should be interpreted cautiously, given the differences in study populations, designs, and screening contexts. Similar findings have been reported in the broader literature, with Dumky et al. reporting lower adherence among African, Asian, and Eastern European women [47].

These variations in screening participation may be related to differences in the organization of the healthcare system, insurance coverage, and migrants’ access to healthcare services in the area where each study was conducted. These differences may also reflect existing social and healthcare disparities based on socioeconomic and cultural factors associated with mammography adherence.

To conclude, the review findings highlight the complex interaction of factors (individual, social, and cultural) that shape women's behavior toward breast cancer screening. Adherence appears to be associated not only with knowledge, perceived risk, and benefits, but also with access to healthcare services, social support, and cultural and religious values. As a practical implication of these findings, enhancing healthcare professionals’ transcultural competence and developing targeted culturally tailored interventions may help address barriers and support women's participation in screening programs.

Based on the findings of this review, multidimensional and culturally appropriate interventions that respect women’s beliefs and cultural diversity may improve mammography adherence. Such strategies could include empowering women, strengthening social support networks, providing screening appointment reminders, and reducing financial barriers through free screening services. However, their effectiveness should be further evaluated in appropriately designed future interventional studies.

Limitations

Several limitations of this review need to be considered when interpreting the findings. Most of the included studies were cross-sectional and relied on self-reported data. This methodology may limit the generalizability of the findings, as self-reported data are subject to recall and reporting biases. Due to the predominance of cross-sectional designs among the included studies, causal relationships between the factors and screening adherence cannot be determined.

Another limitation was that one study assessed the reliability and validity of a measurement tool rather than directly assessing screening adherence, possibly limiting its comparability. However, it provided valuable insights regarding cultural beliefs, perceptions, and perceived barriers, all of which are central concepts to understanding adherence. In addition, the methodological quality of this psychometric study was not formally assessed, as no alternative quality appraisal tool was applied. This limitation should be considered when interpreting its findings.

The included populations in the review originated from different countries and different cultural backgrounds and socioeconomic contexts. This heterogeneity may limit direct comparisons across studies as well as the generalization of findings. Furthermore, the inclusion of only English-language studies published between 2015 and 2025 may have led to the exclusion of other relevant studies published in other languages or before 2015.

An additional limitation concerns the use of studies involving only native women for comparison in the discussion. These studies were not included in the systematic review but were cited to compare the review findings. However, these studies were mainly conducted in countries of Asia and Africa and provided additional insights about cultural beliefs, religious perceptions, and fatalism. Nevertheless, caution is required when interpreting these comparisons, as findings from native populations may not fully reflect specific conditions and experiences of migrant women.

Despite these limitations, this systematic review provides an analytical overview of factors associated with adherence to mammography screening among women from diverse cultural backgrounds.

## Conclusions

This systematic review highlighted the complex and multifaceted factors associated with women's adherence to mammography screening. Factors associated with greater adherence included educational level, health insurance coverage, supportive social and family networks, and clinical advice and guidance from healthcare professionals. In contrast, financial difficulties, language and communication barriers, cultural perceptions and fatalistic beliefs, as well as examination discomfort, were associated with lower adherence. Analysis of these findings showed variations across countries and populations. These geographical variations highlight existing health inequities and the importance of policy commitment, targeted actions, and culturally tailored interventions. Based on the review findings, multilevel and culturally tailored interventions adapted to the specific populations may be beneficial in addressing barriers to mammography adherence and should be further evaluated.

At the same time, as a practical implication of the review findings, enhancing healthcare professionals’ cultural competence may support the provision of equitable care and strengthen health education and health promotion among culturally diverse populations. To further understand the relationship between mammography screening and factors associated with adherence, future research should focus on the systematic evaluation of culturally tailored interventions and strategies through longitudinal study designs, long-term follow-up of populations, and assessment of their effectiveness. This may contribute to the development of more effective prevention and public health strategies.

## References

[1] Bray F, Laversanne M, Sung H, Ferlay J, Siegel RL, Soerjomataram I, Jemal A (2024). Global cancer statistics 2022: GLOBOCAN estimates of incidence and mortality worldwide for 36 cancers in 185 countries. CA Cancer J Clin.

[2] (2024). World Health Organization: Breast cancer. https://www.who.int/news-room/fact-sheets/detail/breast-cancer.

[3] (2024). American Cancer Society (ACS): How common is breast cancer. https://www.cancer.org/cancer/types/breast-cancer/about/how-common-is-breast-cancer.html..

[4] (2023). Canadian Cancer Society: Canadian Cancer Statistics 2022: a special report on cancer prevalence. https://cancer.ca/en/cancer-information/cancer-types/breast/statistics.

[5] Cancer Institute NSW (2024). Cancer Institute NSW: Breast cancer screening. https://www.cancer.nsw.gov.au/prevention-and-screening/screening-and-early-detection/breast-cancer-screening.

[6] Banks J, Wyld L (2023). Epidemiology of Breast Cancer. Breast Cancer Management for Surgeons.

[7] Winters S, Martin C, Murphy D, Shokar NK (2017). Breast cancer epidemiology, prevention, and screening. Prog Mol Biol Transl Sci.

[8] (2026). World Health Organization: World health statistics. https://www.who.int/publications/i/item/9789241507936.

[9] US Preventive Services Task Force (USPSTF (2026). US Preventive Services Task Force (USPSTF):Breast cancer: screening. https://www.uspreventiveservicestaskforce.org/uspstf/recommendation/breast-cancer-screening..

[10] (2026). European Commission: European guidelines on breast cancer screening and diagnosis. http://care.jrc.ec.europa.eu/en/ecibc/european-breast-cancer-guidelines.

[11] Nguyen AT, Duckworth ED, Li RA, Galiano RD (2026). Disparities in breast cancer screening, diagnosis, and outcomes among Vietnamese American women: a systematic review. J Surg Oncol.

[12] Nassur J, Dajee D, Leader A, DiSantis K (2025). Barriers to breast, cervical, and colorectal cancer screenings faced by refugees resettled in the United States: a rapid review. J Immigr Minor Health.

[13] Sheppard VB, Hurtado-de-Mendoza A, Song M, Hirpa F, Nwabukwu I (2015). The role of knowledge, language, and insurance in endorsement of cancer screening in women of African origin. Prev Med Rep.

[14] Roh S, Burnette CE, Lee YS, Jun JS, Lee HY, Lee KH (2018). Breast cancer literacy and health beliefs related to breast cancer screening among American Indian women. Soc Work Health Care.

[15] Hippman C, Moshrefzadeh A, Lohn Z (2016). Breast cancer and mammography screening: knowledge, beliefs and predictors for Asian immigrant women attending a specialized clinic in British Columbia, Canada. J Immigr Minor Health.

[16] March S, Villalonga B, Sanchez-Contador C, Vidal C, Mascaro A, Bennasar ML, Esteva M (2018). Barriers to and discourses about breast cancer prevention among immigrant women in Spain: a qualitative study. BMJ Open.

[17] Freund A, Cohen M, Azaiza F (2019). Factors associated with routine screening for the early detection of breast cancer in cultural-ethnic and faith-based communities. Ethn Health.

[18] Kwok C, Tranberg R, Lee FC (2015). Breast cancer knowledge, attitudes and screening behaviors among Indian-Australian women. Eur J Oncol Nurs.

[19] Ogunsiji OO, Kwok C, Fan LC (2017). Breast cancer screening practices of African migrant women in Australia: a descriptive cross-sectional study. BMC Womens Health.

[20] Kwok C, Endrawes G, Lee CF (2016). Breast cancer screening beliefs questionnaire: psychometric properties assessment of the Arabic version. Eur J Oncol Nurs.

[21] Pilkington L, Haigh MM, Durey A, Katzenellenbogen JM, Thompson SC (2017). Perspectives of Aboriginal women on participation in mammographic screening: a step towards improving services. BMC Public Health.

[22] Suwankhong D, Liamputtong P (2018). Early detection of breast cancer and barrier to screening programmes amongst Thai migrant women in Australia: a qualitative study. Asian Pac J Cancer Prev.

[23] Gill M, Cohen-Cline H, Holtorf M, Vartanian K (2023). Mammogram perceptions, communication, and gaps in care among individuals with non-English language preference in Oregon and Washington states. Prev Med Rep.

[24] Miller BC, Sarma EA, Sun Y, Messina CR, Moyer A (2020). Psychosocial predictors of mammography history among Chinese American women without a recent mammogram. Ethn Health.

[25] Kohler RE, Dharamdasani T, Tarn J (2025). Breast cancer beliefs and screening behaviors among South Asian immigrant women living in the United States. BMC Womens Health.

[26] Saadi A, Bond BE, Percac-Lima S (2015). Bosnian, Iraqi, and Somali refugee women speak: a comparative qualitative study of refugee health beliefs on preventive health and breast cancer screening. Womens Health Issues.

[27] Gorman DR, Porteous LA (2018). Influences on Polish migrants' breast screening uptake in Lothian, Scotland. Public Health.

[28] Damiani G, Federico B, Basso D (2012). Socioeconomic disparities in the uptake of breast and cervical cancer screening in Italy: a cross sectional study. BMC Public Health.

[29] Rasu RS, Rianon NJ, Shahidullah SM, Faisel AJ, Selwyn BJ (2011). Effect of educational level on knowledge and use of breast cancer screening practices in Bangladeshi women. Health Care Women Int.

[30] Mohammadnabizadeh S, Farkhani EM, Talkhi N (2023). Predictive factors of breast cancer mammography screening among Iranian women. J Prev Med Hyg.

[31] Ebrahimoghli R, Aghaei MH, Azami-Aghdash S, Houssami N (2025). Uptake of breast cancer screening practices in low- and middle-income countries: a systematic review and meta-analysis. J Natl Cancer Inst.

[32] Abdou A, Van Hal G, Dille I (2020). Awareness, attitudes and practices of women in relation to breast cancer in Niger. Heliyon.

[33] Halmata M, Tagne Simo R, Nganwa Kembaou G (2021). Breast cancer awareness and screening practice amongst health personnel and general population of the littoral region of Cameroon. Heliyon.

[34] Elias-Rizk T, Issa E, Ammanouil E, Abou Khalil M, Salameh P, Abi-Gerges A (2024). Breast cancer screening in Lebanon: understanding knowledge, attitudes and barriers. Clin Epidemiol Glob Health.

[35] Chandra M, Yu R, Shete S (2023). Association between employer-based health promotion programs and adherence to breast cancer screening in Texas. Prev Med Rep.

[36] Racine L, Isik Andsoy I (2022). Barriers and facilitators influencing Arab Muslim immigrant and refugee women's breast cancer screening: a narrative review. J Transcult Nurs.

[37] Ponce-Chazarri L, Ponce-Blandón JA, Immordino P, Giordano A, Morales F (2023). Barriers to breast cancer-screening adherence in vulnerable populations. Cancers (Basel).

[38] Wu TY, Chen SL (2017). Breast cancer screening practices and related health beliefs among Taiwanese nurses. Asia Pac J Oncol Nurs.

[39] Georga D, Zartaloudi A, Saridi M (2025). Factors determining women's attitudes and knowledge toward cancer screening: a systematic review. Healthcare (Basel).

[40] Jamal J, MacMillan F, McBride KA (2021). Barriers and facilitators of breast cancer screening amongst culturally and linguistically diverse women in South Western Sydney: a qualitative explorative study. Int J Environ Res Public Health.

[41] Tavakoli B, Feizi A, Zamani-Alavijeh F, Shahnazi H (2024). Factors influencing breast cancer screening practices among women worldwide: a systematic review of observational and qualitative studies. BMC Womens Health.

[42] Omisore AD, Odedeyi AA, Famurewa OC (2022). Practice, perceptions, and prospects of mammography screening in Nigeria: insights from a national survey of female health workers. Clin Breast Cancer.

[43] Rauch JI, Daniels J, Robillard A, Joseph RP (2024). Breast cancer screening among African immigrants in the United States: an integrative review of barriers, facilitators, and interventions. Int J Environ Res Public Health.

[44] Al-Hanawi MK, Hashmi R, Almubark S, Qattan AM, Pulok MH (2020). Socioeconomic inequalities in uptake of breast cancer screening among Saudi women: a cross-sectional analysis of a national survey. Int J Environ Res Public Health.

[45] Momenimovahed Z, Tiznobaik A, Taheri S, Hassanipour S, Salehiniya H (2020). A review of barriers and facilitators to mammography in Asian women. Ecancermedicalscience.

[46] Bhargava S, Hofvind S, Moen K (2019). Gender, letters, relatives, and God: mediating actors in mammographic screening among Pakistani women in Norway. Acta Radiol Open.

[47] Dumky H, Fridell K, Leifland K, Metsälä E (2023). Breast cancer screening and immigrant women-a scoping review of attendance, knowledge, barriers and facilitators. Nurs Open.

[48] Sathiyaseelan G, Hashim SM, Nawi AM (2025). Sociocultural factors influencing women's adherence to colorectal, breast, and cervical cancer screening: a systematic review. BMC Public Health.

[49] Alanazi MF, Thirunavukkarasu A, Alrowily M (2023). A cross-sectional evaluation of knowledge about breast cancer and perceived barriers to the uptake of mammogram screening among Northern Saudi women: a population-based study. Breast Cancer (Dove Med Press).

[50] Bao Y, Kwok C, Lee CF (2017). Psychometric properties of the modified breast cancer screening beliefs questionnaire among Mainland Chinese women. Eur J Oncol Nurs.

